# Type 2 Diabetes Mellitus Patients’ Knowledge About Disease Complications and Management Targets for Glucose, Lipids, Blood Pressure, and Body Weight

**DOI:** 10.7759/cureus.62766

**Published:** 2024-06-20

**Authors:** Haider A Alidrisi, Mahdi M Hammoud, Hasan Y Abd Ali, Mohammed E Radhi

**Affiliations:** 1 Diabetes and Endocrinology, Faiha Specialized Diabetes, Endocrine, and Metabolism Center, Basrah, IRQ; 2 Diabetes and Endocrinology, University of Basrah, College of Medicine, Basrah, IRQ; 3 Medicine, University of Basrah, College of Medicine, Basrah, IRQ

**Keywords:** type 2 diabetes, blood pressure target, lipid target, glucose target, patient knowledge, patient knowledge

## Abstract

Background

As a result of the chronic nature of type 2 diabetes mellitus (T2DM), its complications, and treatment complexity, patients should have a comprehensive knowledge of various aspects of T2DM management and follow-up. The study aimed to assess T2DM patients’ knowledge of disease complications and their screening strategies and the management targets for glucose, lipids, blood pressure, and body weight.

Methods

This was a cross-sectional and questionnaire-based study including 205 adult patients with T2DM from November 2023 to March 2024. The patients were randomly selected at one tertiary endocrine center and the outpatient clinics of three teaching hospitals in Basrah, southern Iraq. Social and disease-related data were collected. Another 18 T2DM-related questions were designed to assess the patients’ knowledge about the aim of treatment; T2DM complications and their screening; the recommended targets for glycemic, lipid, and blood pressure control; and the recommended exercise and weight loss. We gave one point for each correct answer and considered a final score of 10/18 as adequate.

Results

Of a total of 205 patients, 109 (53.2) were women. The mean age for patients was 48.7 ± 13.1 years. Based on the patients’ responses, 107 (52.2%) had adequate knowledge about T2DM. Questions about the target fasting and post-prandial capillary blood glucose, HbA1c target and frequency, and naming the current treatment were the most correctly answered questions (80.9%, 73.1%, 68.7%, and 72.6%, respectively). Questions about the lipid target, definition of hypoglycemia, and ideal lifestyle for T2DM (exercise and bodyweight loss) were least correctly answered. Patients younger than 40 years old, being a man, with a higher educational level, and T2DM duration of more than five years had significantly higher T2DM knowledge.

Conclusions

Only half of the patients had adequate T2DM knowledge. Better degree of knowledge was particularly observed in patients with younger age, male gender, higher educational level, and longer T2DM duration. There is a need to promote diabetes education strategies for people with T2DM.

## Introduction

Type 2 diabetes mellitus (T2DM) represents the major type of diabetes around the world. It is caused by a complex pathogenesis (ominous octet) with two main anomalies, insulin resistance and progressive decline in the β-cell function [1,2]. The clinical manifestations of T2DM related to hyperglycemia are often less dramatic as compared to type 1 diabetes mellitus (T1DM). This results in a progressive silent disease until disease-related chronic complications develop [3]. Over the past decades, T2DM experienced an increase in its prevalence all over the world, including the Middle East and North Africa region, with an expected rise in the prevalence by 110% in 2045 [4]. In Iraq, the estimated prevalence of T2DM ranges between 8.5 and 13.9% [5]. Moreover, in a local study in Basrah, southern Iraq, the age-adjusted prevalence of T2DM was 19.7% (11% found on active screening) [6].

T2DM represents a global emergency due to the associated wide range of diabetes-related complications. Cardiac failure, ischemic heart disease, stroke, diabetic nephropathy, diabetic retinopathy, diabetic neuropathy, limb amputations, and death were the endpoint complications of diabetes, which can be prevented by health education, preventive strategies, and early effective management of hyperglycemia and the associated cardiovascular risk factors [7]. Studies in Iraq suggest that the rates of these complications and their risk factors are underestimated by the absence of effective active screening [8,9].

The international and local guidelines recommended a multifactorial approach to diabetes management [10-12]. The glucose control is represented by hemoglobin A1c (HbA1c) less than 7%, fasting blood glucose (FBG) 80-130 mg/dL, postprandial blood glucose (PBG) less than 180 mg/dL, and avoidance of hypoglycemia with blood glucose less than 70 mg/dL. Blood pressure control is represented by a target of less than 130/80 mmHg. Dyslipidemia management by targeting the low-density lipoprotein cholesterol (LDL) less than 100 mg/dL (<70 mg/dL in high cardiovascular risk) and triglyceride (TG) less than 150 mg/dL. The final approach includes individualized lifestyle by diet and exercise and smoking cessation.

Unfortunately, the vast majority of T2DM patients had uncontrolled glycemia despite the set goal for the prevention and control of T2DM in Iraq [13]. Security and political challenges make this goal hard to reach [5]. Relatively low healthcare funds used to be distributed for diabetes management plans [14]. Fitness and exercise opportunities are still lacking in Iraq, particularly for women [15]. Despite the development of programs for early detection, primary care, and education of chronic diseases like T2DM and hypertension, their implementation by primary health care was not successful. Hence, diabetes healthcare relies mainly on secondary care, tertiary care, and private sectors [16].

Based on the chronic nature of T2DM and its complications, its high prevalence in Iraq, and the complexity of treatments, the management of the disease represents a major challenge to the health system. To give high-quality T2DM care, patients should have a comprehensive knowledge of the aim of T2DM treatment, treatment strategies (medications and lifestyle), T2DM chronic complications screening, hypoglycemia, and treatment targets in the form of hemoglobin A1c (HbA1c), serial monitoring of home blood glucose, blood pressure, and dyslipidemia [17-20].

In this study, we aimed to assess T2DM patients’ knowledge of disease complications and their screening strategies and the management targets for glucose, lipids, blood pressure, and body weight.

## Materials and methods

This is a cross-section study including adult patients with T2DM seen at Faiha Specialized Diabetes Endocrine and Metabolism Center (FDEMC) and the outpatient clinics of Basrah Teaching Hospital, Al-Sader Teaching Hospital, and Al-Mawani Teaching Hospital) in Basrah, southern Iraq. The study period was from November 2023 to March 2024.

Inclusion criteria were adult patients aged equal to or more than 18 years, with T2DM that was diagnosed one or more years ago. We excluded patients with no interest or were not willing to participate in the study.

Data collection

Personal and disease-related data were collected from each patient. Personal data were in the form of age, gender, marital status, and level of education. The disease-related data were in the form of T2DM duration, the presence of any diabetes-related complication (cardiovascular disease, diabetic nephropathy, diabetic retinopathy, diabetic neuropathy, and diabetic foot), previous hypoglycemia, hypertension, and dyslipidemia.

To test the patients’ basic knowledge about T2DM, we formulated a questionnaire that included 18 diabetes-related simple questions (one point was given for every correct answer), as shown in Table 1. We tried to focus on the patient’s knowledge of T2DM by choosing a series of questions that are commonly discussed with the patients during clinical interviews and covered the multifactorial cardiovascular approach in the management of T2DM.

**Table 1:** Eighteen type 2 diabetes knowledge questions

Questions:	Correct	Wrong
Name your current drugs for diabetes?	1	0
Correctly names his current glucose lowering medications.		
Why do you need to control your blood glucose?	1	0
To prevent the diabetes-related complications.		
What are the chronic complications of diabetes?	1	0
The patient should tell at least two (cardiovascular diseases, diabetic nephropathy, diabetic retinopathy, diabetic neuropathy, and diabetic foot).		
How frequent is needed check your eye by ophthalmologist?	1	0
At least once yearly.		
How frequent is needed to check renal function (creatinine and/or urine albumin-creatinine ratio)?	1	0
At least once yearly.		
Do you self-examine your feet?	1	0
Yes, daily.		
What is hemoglobin A1c and its target?	1	0
Represent the degree of glycemic control for last three months and should be targeted to less than 7%.		
How often hemoglobin A1c should be measured?	1	0
Two to four times annually.		
How frequent you measure home capillary blood glucose?	1	0
Patients treated with insulin: ≥ 2 times daily and before injection of insulin. Patients treated with oral agents: 2 times weekly (more frequently if poorly controlled or upon doctor advice).		
What is the target fasting capillary blood glucose?	1	0
80-130 mg/dL.		
What is the target post-prandial capillary blood glucose?	1	0
Less than 180 mg/dL.		
What is hypoglycemia?	1	0
Less than 70 mg/dL.		
What is the target low-density lipoprotein (LDL-C)?	1	0
Less than 100 mg/dL or lower upon doctor advice.		
What is the target triglyceride (TG)?	1	0
Less than 150 mg/dL.		
What is the recommended target blood pressure?	1	0
Less than 130/80 mmHg.		
Do you need to reduce your bodyweight?	1	0
Yes.		
How much bodyweight loss is needed for diabetes?	1	0
5% of the bodyweight.		
What is the recommended exercise for diabetes?	1	0
Regular moderate-intensity exercise like brisk walking or running of three to five sessions/week (150 min).		
Total points	18	

We considered the patients who scored 10 points out of 18 as having adequate knowledge about T2DM, while patients with a score of less than 10 were considered as having inadequate knowledge about T2DM. 

Statistical analysis

The sample size for the study was calculated to be 196 patients with T2DM. This was done using Cochran’s formula based on population proportions of T2DM of 15%, confidence interval of 95% (z-score 1.96), and alpha value of 0.05. The collected data were analyzed by the IBM SPSS Statistics for Windows, version 26.0 (released 2019, IBM Corp., Armonk, NY). Categorical variables were summarized as numbers (N) and percentages (%). Continuous variables were summarized as mean ± standard deviation (M ± SD). The chi-square test was used to determine the relationship between variables. A P-value of <0.05 was defined as statistical significance.

## Results

Table 2 summarizes the general characteristics of the study patients. Of a total of 205 patients, 109 (53.2) were women. The mean age for patients was 48.7 ± 13.1 years, and the mean T2DM duration was 7.3 ± 4.2 years. The patients’ reported hypoglycemia rate was 57.6%, and chronic T2DM complications were presented in 47.8% of the patients. Hypertension and dyslipidemia were presented in 56.6% and 51.7% of the patients, respectively.

**Table 2:** Baseline general characteristics of the study patients (N = 205) *Patient documented hypoglycemia. µ cardiovascular disease, diabetic nephropathy, diabetic retinopathy, diabetic neuropathy, and diabetic foot. Abbreviations: SD, standard deviation; N, number; T2DM, type 2 diabetes mellitus.

Variable		Mean ± SD or N (%)
Mean age (years)		48.7 ± 13.1
Age range (years)		19 - 70
Gender (women)		109 (53.2)
Marital status	Single	26 (12.7)
	Married	118 (57.6)
	Divorced	29 (14.1)
	Widow	32 (15.6)
Education	Primary	74 (36.1)
	Secondary	64 (31.2)
	College	67 (32.7)
T2DM duration (years)		7.3 ± 4.2
T2DM duration range (years)		1 - 25
	< 5 years	60 (29.3)
	5-10 years	95 (46.3)
	>10 years	50 (24.4)
Hypoglycemia*		118 (57.6)
Any chronic complication of T2DM^µ^		98 (47.8)
Hypertension		116 (56.6)
Dyslipidemia		106 (51.7)

**Figure 1:**
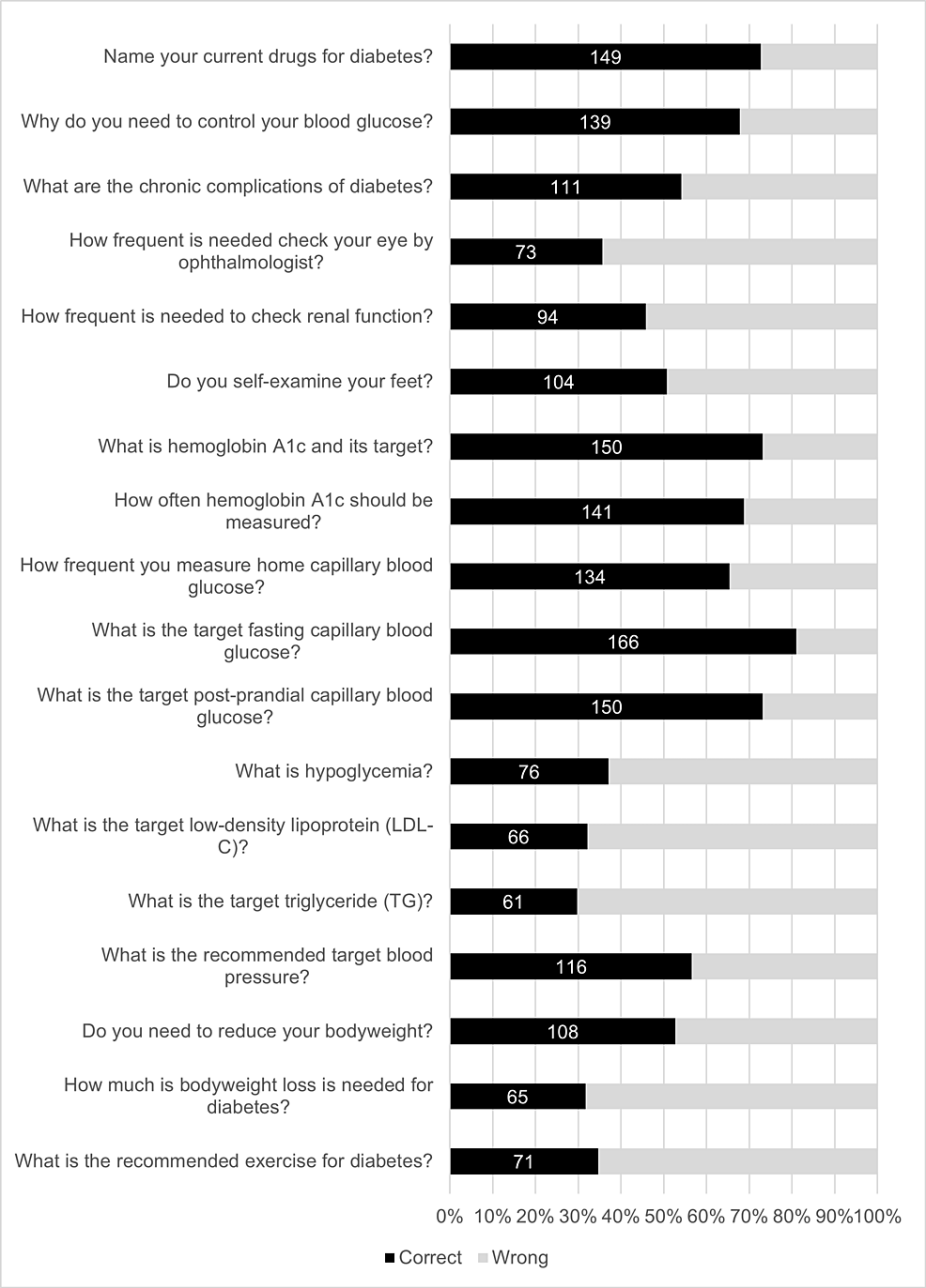
Study patients’ responses to the diabetes knowledge questions

As shown in Figure 1, the patients had variable responses to the diabetes-related knowledge questions. Questions about the target fasting and post-prandial capillary blood glucose, HbA1c target and frequency, and naming the current treatment were the most correctly answered questions (80.9%, 73.1%, 68.7%, and 72.6%, respectively). Questions about the lipid target, definition of hypoglycemia, and ideal lifestyle for T2DM (exercise and bodyweight loss) were least correctly answered.

The mean final score for the patients was 9.6 ± 3.1 points, a median of 10 points, and only 107 (52.2%) had a score of equal to or more than 10, as shown in Table 3.

**Table 3:** Overall results of the patients’ responses to diabetes knowledge questions

Score statistics	Results
Mean score (points)	9.6 ± 3.1
Median score (points)	10
Score range (points)	1-18
Score ≥ 10/18 N(%)	107 (52.2)

Patients younger than 40 years old, being a man, with a higher educational level, and T2DM duration of more than five years had significantly higher T2DM knowledge (69.1%, 60.4%, 67.2%, and 57.9% respectively), as shown in Table 4.

**Table 4:** Age, gender, educational level, T2DM duration, and the presence of any T2DM chronic complications' effect on the patients’ knowledge about T2DM *Chi-square test. µ cardiovascular disease, diabetic nephropathy, diabetic retinopathy, diabetic neuropathy, and diabetic foot. Abbreviations: SD, standard deviation; N, number; T2DM, type 2 diabetes mellitus.

Variables		Score ≥ 10/18	Score < 10/18	P value*
		N (%)	N (%)	
Age	<40 years	38 (69.1)	17 (30.9)	0.01
	40-59 years	41 (47.7)	45 (52.3)	
	≥60 years	28 (43.8)	36 (56.3)	
Gender	Men	58 (60.4)	38 (39.6)	0.02
	Women	49 (45.0)	60 (55.0)	
Education	Primary	27 (36.5)	47 (63.5)	0.001
	Secondary	35 (54.7)	29 (45.3)	
	College	45 (67.2)	22 (32.8)	
DM duration	<5 years	23 (38.3)	37 (61.7)	0.03
	5-10	55 (57.9)	40 (42.1)	
	>10 years	29 (58.0)	21 (42.0)	
Any chronic complication of T2DM^µ^	Yes	43 (43.9)	55 (56.1)	0.28
	No	55 (51.4)	52 (48.6)	

## Discussion

This was a community-based questionnaire-based study that assessed T2DM patients’ knowledge about T2DM complications and their screening strategies and the management targets for glucose, lipids, blood pressure, and body weight. In general, half of the patients had adequate knowledge. They were more likely to know about the glucose-centric approach of T2DM management (their glucose-lowering medications, HbA1c, FBG, and PPG). Meanwhile, information about other aspects in the form of hypoglycemia, diabetes-related complications, and the recommended lifestyle measures were less known by the patients.

Many studies have been done with a similar aim to assess patients' knowledge of T2DM using different questionnaires and on patients from various cultural backgrounds. These resulted in a variability in the overall degree of knowledge found in these studies.

In a study that was done in Saudi Arabia, 42% had excellent knowledge about T2DM using a questionnaire that assessed the general information about T2DM. Increasing age and working in the medical field were the factors that were associated with better knowledge about T2DM. Meanwhile, gender, income, and level of education did not affect the patients’ knowledge [21]. Another finding from Thailand found a general knowledge of about 71%, and having diabetes-related complications and a higher educational level were associated with better knowledge. However, there were no gender differences in the knowledge [22]. In the present study, being a man, younger age, and higher education level were associated with better knowledge. These differences may be explained by the different focus in our questions that were designed to assess the knowledge about the management and follow-up plans.

In an Indian study by Gulabani et al. [23], the average knowledge about diabetes-related complications was 70% and more likely in men, which is higher than our study. However, in this study, only 6%, 20%, and 40% knew about HbA1c, the targets for FBG, and PPG, respectively. Only 50% knew that renal function should be checked, 40% knew about regular feet checking, and 30% knew about weight loss. While a higher knowledge about hypoglycemia was seen at about 50%, the questioning was about symptoms rather than the level of blood glucose like in our study (57.6% of patients report hypoglycemia, and only 37.0% correctly defined hypoglycemia). Other data from Sharma et al. showed a degree of knowledge of 50% with a higher degree of knowledge seen among men and those with higher educational levels [24]. In a study from South Africa, about 67% of patients passed the diabetes knowledge test, and the best results were seen among women, middle-aged, and longer T2DM duration [25]. These findings are comparable to the results of the present study apart from the reverse in the gender results. Despite the equal gender proportions in the present study and the equal gender prevalence of T2DM [6], men tended to have better knowledge about T2DM. These findings may be explained by the gender-based bias in the health-seeking behavior that was reported in different studies [26,27].

Better knowledge about the aspects of T2DM by the patients may aid in the care of the disease and its complications. Some studies found a positive correlation between the patients’ knowledge and the glycemic control [28,29]. While other studies did not find a significant correlations [24,30]. Unfortunately, our study did not include an analysis of the correlation between the patients’ knowledge and glycemic control. This is one limitation, besides the absence of dietary-based questions in the study questionnaire. However, in the present study, we tried to assess the patients' knowledge of T2DM by choosing a series of questions that are commonly discussed with patients during clinical interviews and covered the multifactorial cardiovascular approach in the management of T2DM.

## Conclusions

Only half of the patients with T2DM had adequate knowledge about T2DM. Social characteristics were correlated with the patients’ knowledge in which younger age, men, higher educational level, and longer T2DM duration had better knowledge. These results underline the importance of developing policies that prioritize health education for people with T2DM. We recommend the improvement of our patients’ disease awareness by encouraging the provision of basic T2DM education in the primary, secondary, and tertiary sectors and other different channels like television and social media.
